# The Impact of Spatial Scales and Spatial Smoothing on the Outcome of Bayesian Spatial Model

**DOI:** 10.1371/journal.pone.0075957

**Published:** 2013-10-11

**Authors:** Su Yun Kang, James McGree, Kerrie Mengersen

**Affiliations:** 1 Mathematical Sciences School, Queensland University of Technology, Brisbane, Queensland, Australia; 2 Cooperative Research Centre for Spatial Information, Melbourne, Victoria, Australia; Arizona State University, United States of America

## Abstract

Discretization of a geographical region is quite common in spatial analysis. There have been few studies into the impact of different geographical scales on the outcome of spatial models for different spatial patterns. This study aims to investigate the impact of spatial scales and spatial smoothing on the outcomes of modelling spatial point-based data. Given a spatial point-based dataset (such as occurrence of a disease), we study the geographical variation of residual disease risk using regular grid cells. The individual disease risk is modelled using a logistic model with the inclusion of spatially unstructured and/or spatially structured random effects. Three spatial smoothness priors for the spatially structured component are employed in modelling, namely an intrinsic Gaussian Markov random field, a second-order random walk on a lattice, and a Gaussian field with Matérn correlation function. We investigate how changes in grid cell size affect model outcomes under different spatial structures and different smoothness priors for the spatial component. A realistic example (the Humberside data) is analyzed and a simulation study is described. Bayesian computation is carried out using an integrated nested Laplace approximation. The results suggest that the performance and predictive capacity of the spatial models improve as the grid cell size decreases for certain spatial structures. It also appears that different spatial smoothness priors should be applied for different patterns of point data.

## Introduction

Spatial data are available in various forms; at point level, grid level or area level. In the context of epidemiological studies, area level data are usually utilized due to its availability. This is because some phenomena are expressed naturally as area level data such as contextual variables in social epidemiology. In addition, disease incidence is often aggregated to administrative districts in order to protect patient confidentiality. For convenience, the aggregated data are further used to study small-scale geographical variation. Consequences of this practice include loss of individual information and potential ecological fallacy [1], where the latter refers to the difference between individual and group level estimates of risk measures. The aggregated data may also suffer from changes in geographical boundaries over time which calls into question the value of any analyses. Another problem concerning the aggregated data is the modifiable area unit problem, which is defined as sensitivity of statistical results to the definition of geographical units over which data are collected [2]. For instance, various datasets may exhibit different spatial patterns when viewed at one spatial scale compared to another, which is known as a ‘scale’ effect [3].

In contrast, point level disease data contain desirable individual information and precise domicile addresses in some instances, alleviating the issue of ecological bias. However, they are often difficult to access due to confidentiality issues. Even if they are available, patients' residential locations have to be protected and are not allowed to be published, which has restricted the types of analyses that can be carried out on point level disease datasets. Another limitation is that the study of small-scale geographical variation is not practicable if using individual level disease data. As a compromise, we utilize point level disease data in this study but employ a grid level modelling approach to study the geographical variation of residual disease risk using regular grid cells. As a result, the issue of patient confidentiality and ecological bias are both addressed in this study.

We model the individual disease risk using a logistic model with the inclusion of spatially unstructured and/or spatially structured random effects. Geographical variation of residual disease risk is modelled using a spatial component that allows for the heterogeneity of random effects and borrows strength from neighboring grid cells. The grid cells are far smaller than the typical administrative districts and therefore allow for better specification and identification of spatial random effects. Many ecological responses of interest do not recognize areas or borders defined for administrative purposes, and thus a finer geographical scale of study is often more appropriate for ecological studies [4]. The findings are more relevant and specific to the local population in a finer geographical area.

Despite being less common than studying the geographical variation using the area level data, the grid level modelling approach has rapidly increased in popularity in recent years [5]–[8]. Modelling of disease data at a grid level is a desirable approach as it is geographically more accurate than using area level data and yet protects patient confidentiality. Other advantages include the formation of a generalized linear model and approximation of the covariance structure by a Markov random field, which eases computation [9], [10]. The grid level modelling avoids the need to deal with the problem of changes in administrative boundaries over time. This approach has the flexibility of allowing the spatial scale at which the data are modelled to be manipulated to a practically, biologically, geographically or computationally sensible scale.

One of the challenges in the grid level modelling is the specification of an appropriate spatial scale for a specific spatial dataset. At present, not much is known about the impact of different spatial scales on the outcome of spatial models at different spatial patterns. Without repeating the analyses at multiple scales, it is difficult to know whether the findings at various scales are consistent. According to [11], selection of the spatial scale of analysis should be guided by the purpose of analysis, i.e., whether to draw conclusions at the individual level or the aggregated level. It is thus important to consider analyses at various spatial resolutions in order to identify the most appropriate geographical scale that contributes to significant findings for the problem at hand.

Given this identified challenge, the study has two main aims: (i) to investigate the impact of changes in spatial scale on model outcome for a set of spatial structures; (ii) to evaluate the performance of various Bayesian spatial smoothness priors for spatial dependence, namely an intrinsic Gaussian Markov random field (IGMRF), a second-order random walk (RW2D) on a lattice, and a Gaussian field with Matérn correlation function. Bayesian inference is carried out using integrated nested Laplace approximation (INLA) throughout the study.

We designed a simulation study and utilized a case study to fulfill the aims. The simulated datasets consist of point data with various spatial structures including inhomogeneous point patterns, patterns with local repulsion, patterns with local clustering, and patterns with local clustering in the presence of a larger-scale inhomogeneity. The case study involves the analysis of the Humberside data on childhood leukaemia and lymphoma. This dataset portrays a sparse spatial pattern with potential spatial clustering.

## Methods

### Model

Let *X* be a spatial point-based dataset embedded in an observation window *S*, which is discretized into 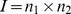 regular grid cells. Let 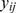 denote the event outcome of the 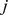-th individual in the 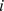-th grid. Here 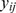 is a binary response that follows a Bernoulli distribution with probability of disease 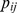. The individual risk, 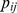, is modelled via the logistic regression model,
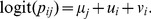
(1)


Spatial variation in the individual risk is modelled using different components including 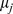, 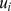, and 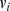, where 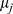 refers to the intercept term for individual 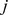, 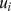 is an unstructured term that accounts for unexplained variability in the model, and 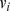 is a spatially structured term that describes the effect of the location by assuming that geographically close areas are more similar than distant areas. In this study, three Bayesian spatial smoothness priors for the spatially structured effect are considered, namely an intrinsic Gaussian Markov random field (IGMRF), a second-order random walk (RW2D) on a lattice [12], and a Gaussian field with Matérn correlation function [13] which includes a range parameter. These three priors are chosen due to their popularity in spatial modelling [14].

### The IID model

The IID model considers a fixed intercept and unstructured random effects 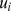.
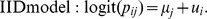



The IID model defines 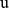 to be a vector of independent, identical and Gaussian distributed random variable (possibly scaled) with mean zero and unknown precision (inverse variance), 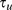:
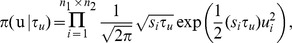



where 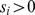 is an optional fixed scale. The precision parameter 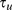 is assigned a Gamma prior.

### The IGMRF model

In the IGMRF model corresponding to (1), the spatially unstructured component, 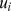, is assumed to be independent and identically distributed (i.i.d.) and normally distributed with mean zero and unknown precision, 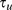. The spatially structured component, 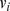, is given an IGMRF prior [12] with unknown precision, 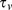. Gamma priors are assigned to the precision parameters 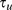 and 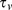.

An IGMRF for 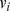 is defined as
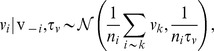



where 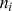 is the number of neighbors of grid cell 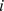, 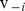 denotes all elements in 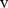 except for 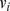, and 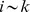 indicates that the two grid cells are neighbors that share a common boundary. A sum-to-zero constraint is imposed on 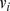 to ensure identifiability of the intercept 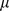. See [12], [15] and [16] for further details. This model has been widely applied in disease mapping to study spatial variation of disease risk [17]–[19]. The neighborhoods in these papers were defined in terms of administrative districts, we consider a finer neighborhood structure in terms of (regular) grid cells, however.

### The RW2D model

The RW2D model corresponding to (1) employs a different formulation for the spatially structured effect. Here, 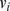 is imposed an RW2D prior on the 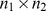 regular lattice, which is alternatively known as a second-order polynomial intrinsic GMRF [12]. This choice is motivated by its application in a discretized log Gaussian Cox process (LGCP) [20]. The model approximates a LGCP only when the grid cells are fine enough.

The RW2D model is defined on a regular grid (see Rue and Held [12], section 3.4.2). The full conditionals of the nodes in the interior (with obvious notation) of the regular grid are as follows
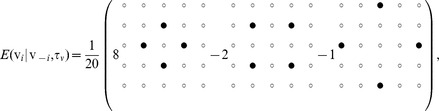


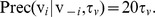



The precision 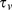 is unknown. As stated by [20], the full conditionals are constructed to mimic the thin plate spline. Corrections to the boundary can be found by using the stencils in [21]. A sum-to-zero constraint is again imposed on the spatial term to ensure identifiability of 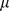. A Gamma prior is assigned to the precision parameter 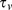.

### The MATERN2D model

The MATERN2D model corresponding to (1) is considered. The spatially structured effect 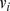 is imposed a prior as a Gaussian field with Matérn correlation function on the 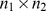 regular lattice. The Matérn isotropic correlation function on an infinite lattice is given as [13], [22], [23],
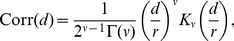



where *d* is the separation distance, 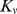 is the modified Bessel function of the second kind of order *v*, 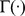 is the Gamma-function, *r* is the range or distance parameter (*r*>0) which measures how quickly the correlations decay with distance, and *v* is the smoothness parameter (*v*>0). The latent field has marginal variance 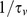 and range *r*. Gamma priors are assigned to both parameters. The Matérn model has great flexibility in modelling the spatial covariance due to the smoothness parameter *v*. A large value of 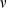 (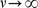) implies a smoother spatial process.

### Computation

In light of the computational cost of Markov chain Monte Carlo (MCMC) methods for spatial inference, we adopt the integrated nested Laplace approximation (INLA) approach proposed by [20]. We note that MCMC might also be possible with desktop computing, but the Laplace approximation is adequate for our purposes. INLA performs approximate Bayesian inference for latent Gaussian models [24], which are defined in three stages as
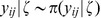
(Observation equation)

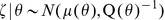
(Latent Gaussian field)

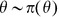
(Parameter model)


where 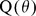 is the precision matrix of the Gaussian random vector 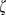, which is sparse. The posterior can be written as
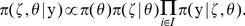



The models considered in this study are regarded as latent Gaussian models by assigning 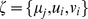, a Gaussian prior with precision matrix 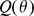. The precision parameters of 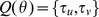 are assigned a prior, Gamma distribution. The desired posterior marginals can be written as
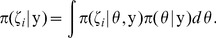
(2)


The vector 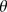 refers to the hyperparameters used in defining prior distributions for the precision of the Gaussian priors. The posterior marginals of 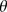 are approximated by
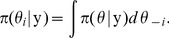
(3)


In order to estimate (2) and (3), nested approximations are constructed, and numerical integration is used to integrate out 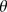. The Laplace approximation to the posteriors of hyperparameters can be written as
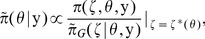
(4)


where 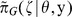 is the Gaussian approximation to the full conditional of 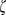 and 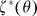 is the mode of the Gaussian approximation for each 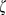. We refer the reader to [20] for more details on INLA computation. See [25]–[29] for Bayesian inference using INLA in various applications. See also [10], [30], [31] on how the approximation of a LGCP on fine grids is carried out using INLA.

Computation in this study is performed in the R package, by calling the inla program. Two steps are taken to run the models. First, the linear predictor of a model is specified using the formula object in R. The specified model can then be run by calling the inla( ) function. We choose “strategy = laplace” to apply a Laplace approximation in (4) to estimate the marginals of the components of the latent field. The output of the inla( ) function generates various statistics such as marginal likelihood, deviance information criterion, effective number of parameters, predictive measures such as logarithmic score [32] and probability integral transform [33], useful to compare and validate models.

As an illustration, the call in R-INLA to fit the IGMRF model is

data  =  list(y, j, region.iid, region.struct)

formula  =  y ∼ –1 + f(j, model = “iid”)

+ f(region.iid, model = “iid”,

hyper = list(theta = list(prior = “loggamma”,param = c(1,0.01))))

+ f(region.struct, model = “besag”, graph  =  “nb5×5.graph”,

hyper = list(theta = list(prior = “loggamma”,param = c(1,0.01))))

result  =  inla(formula, family = “binomial”, Ntrials = 1, data = data, verbose = TRUE,

control.compute = list(dic = TRUE, cpo = TRUE), control.inla = list(strategy = “laplace”))

## Description of Data

### Simulated data

We conducted a simulation study to investigate the impact of spatial scales and spatial smoothing on modelling outcomes. As discussed earlier, a spatial pattern may be present at a given aggregation level and may vanish at other scales. Therefore, using a range of spatial scales, the purpose of this simulation study was to investigate the performance of the models when dealing with different spatial structures of point-based data. We simulated spatial point-based data from various classical point-process models on the unit square. As guided by Illian et al. [31], we considered various scenarios: inhomogeneous point patterns, patterns with local repulsion, patterns with local clustering, and patterns with local clustering in the presence of a larger-scale inhomogeneity. These point-based data include cases and controls which resemble the Bernoulli outcome of an event (or a disease) in practice. We simulated cases and controls from two separate point-process models.

In dataset **X1**, the cases were generated from an inhomogeneous Poisson process with trend function 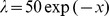 on the unit square. The controls were generated from an inhomogeneous Poisson process with trend function 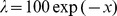 which were then superimposed with the cases. This resulted in point-based data that were inhomogeneously distributed across the space with an average intensity of 107 points per unit square.

Dataset **X2** consisted of cases distributed in patterns with local repulsion, which were generated from a homogeneous Strauss process, with medium repulsion 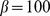 (intensity parameter), interaction parameter 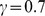 and interaction radius 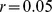, on the unit square. The cases were superimposed with the controls that were generated from an inhomogeneous Poisson process with trend function 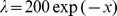. The average intensity of this dataset was 210 points per unit square.

To generate the clustered cases in dataset **X3**, we simulated a homogeneous Thomas process with parameters 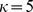 (the intensity of the Poisson process of cluster centers), 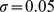 (the standard deviation of the distance of a point from the cluster center) and 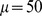 (the expected number of points per cluster), on the unit square. Similarly, the controls were generated from an inhomogeneous Poisson process with trend function 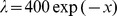. After superimposing the cases and the controls, the average intensity of this dataset was 431 points per unit square.

The cases in dataset **X4** were generated from an inhomogeneous Thomas process with parameters 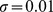 and 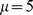 and a simple trend function for the intensity of parent points given by 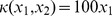, on the unit square. The cases were then superimposed with controls generated from an inhomogeneous Poisson process with trend function 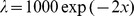. The average intensity of the cases and the controls was 648 points per unit square.

In dataset **X5**, both the cases and controls were generated from an inhomogeneous Poisson process with trend function 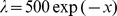 on the unit square. The resulting point-based data were inhomogeneously distributed across the space with an average intensity of 605 points per unit square.

The cases in dataset **X6** were generated from an inhomogeneous Poisson process with trend function 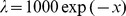 on the unit square. The controls were generated from an inhomogeneous Poisson process with trend function 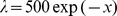 which were then superimposed with the cases. This resulted in point-based data that were inhomogeneously distributed across the space with an average intensity of 982 points per unit square.

**Figure 1:**
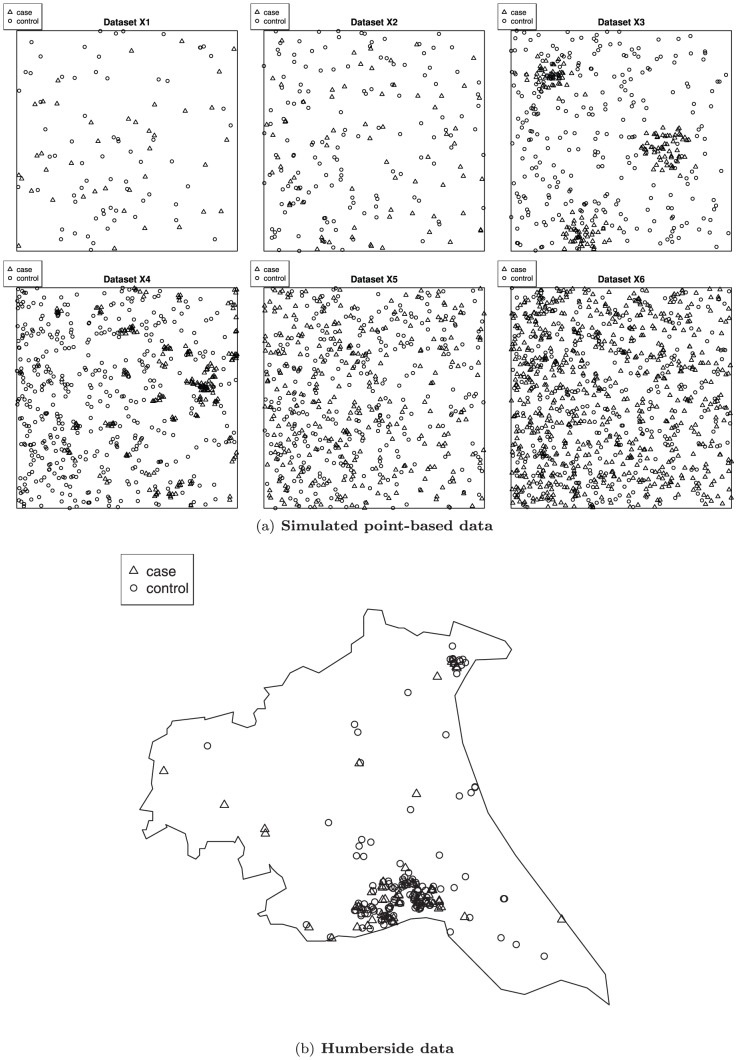
(a) Six patterns of simulated point-based data (top). Various spatial patterns are considered, including inhomogeneous point patterns, patterns with local repulsion, patterns with local clustering, and patterns with local clustering in the presence of a larger-scale inhomogeneity. **(b) The Humberside data on childhood leukaemia and lymphoma** (bottom). The dataset portrays a sparse spatial pattern with a cluster.

Datasets **X1**, **X5** and **X6** were of similar patterns but different degree of denseness. Dataset **X3** had bigger clusters than dataset **X4**. See Figure 1(a) for illustrations of the simulated point-based data.

### Humberside data on childhood leukaemia and lymphoma

We considered a realistic example (Figure 1(b)) available in the spatstat R package to illustrate the four models. The data were first presented and analyzed by [34]. [34] perform the method for detecting spatial clustering of events on this dataset. It is not the aim of this paper to pursue the detection of spatial clusters. We use this dataset as a case study that portrays natural phenomena to investigate the impact of spatial scales and spatial smoothing on modelling outcomes to complement the simulation study. The data contained 62 cases of childhood leukaemia and lymphoma diagnosed in the North Humberside region of England between 1974 and 1986, and 141 controls selected at random from the birth register for the same period. Spatial location of each individual's home address (actually, the centroid for the postal code) was given in the dataset. The dataset had a polygonal observation window; for the analysis, we created a 72.1 km×60.8 km rectangular window to enclose all events.

## Model Fitting and Evaluations

To evaluate the impact of modelling the random effects at different spatial scales, we considered the partitions at the grid level by discretizing the study region using grids 5×5, 10×10, 15×15, 20×20, 25×25, 30×30, 35×35, 40×40, 45×45, and 50×50. The grid 5×5 resulted in 25 grid cells over the unit square, the grid 10×10 resulted in 100 grid cells over the unit square, and so on. So, the grid 5×5 had the largest grid cell size whereas the grid 50×50 had the smallest grid cell size. The cell2nb function in the spdep R package [35] was used to generate a list of neighbors for the grid cells, by applying a queen definition of neighborhood, where two grid cells were termed neighbors if they shared a common edge or vertex. The adjacency matrices were required in the fitting of the IGMRF model.

In terms of prior specification, the precision parameters of the unstructured random effect and spatial effect, 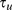 and 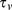, were both assigned Gamma priors with parameters (1,0.01) to impose the same level of spatial smoothing on the spatial field for each model throughout the study. We carried out sensitivity analyses to assess the impact of various choices of prior distributions on the models and found that the influence of priors are negligible on the basis of minimal changes in the deviance information criterion (DIC).

For the purpose of model comparison, DIC was used to select the most parsimonious model after penalizing for model complexity. We note that DIC has been criticized [36], [37] and can be problematic in models with many random effects [38]. Though it fails in some contexts, the use of DIC is appropriate in most generalized linear modelling problems and is a popular Bayesian model choice criterion for comparing complex hierarchical models [39]. A smaller DIC indicates a better fit of the model. As suggested by [39], DIC should not be used as an absolute measure of the ‘best’ model, but rather a method for screening alternative formulations in order to provide an indication of the relative fit of a set of candidate models. Candidate models receiving DIC within 1–2 of the ‘best’ deserve consideration, while 3–7 have considerably less support [39].

The logarithmic score (LS) for each model was also computed [32] to assess the predictive performance of these models. Each model was assigned a numerical score based on the predictive distribution using the cross-validated scoring rules. For discrete observations 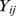, the LS is defined as
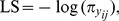



where 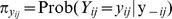 denotes the cross-validated predictive probability mass at the observed event. A smaller LS indicates a better predictive power of the model [40], [41].

## Results

**Figure 2:**
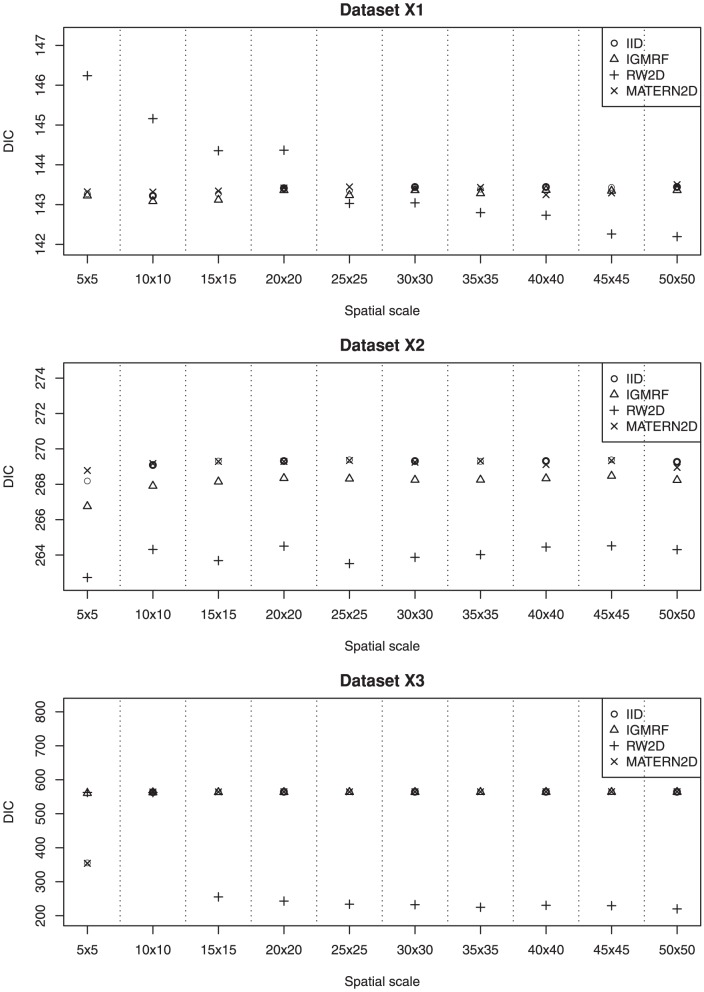
The estimated DIC of the four models for datasets X1, X2 and X3 at various spatial scales. The RW2D model fitted at small grid cell sizes appears to be a reasonable choice for dataset **X1**. For dataset **X2**, the RW2D model produces the smallest DIC at all spatial scales. The RW2D model also performs better than the three other models at grids 15×15 and above.

**Figure 3:**
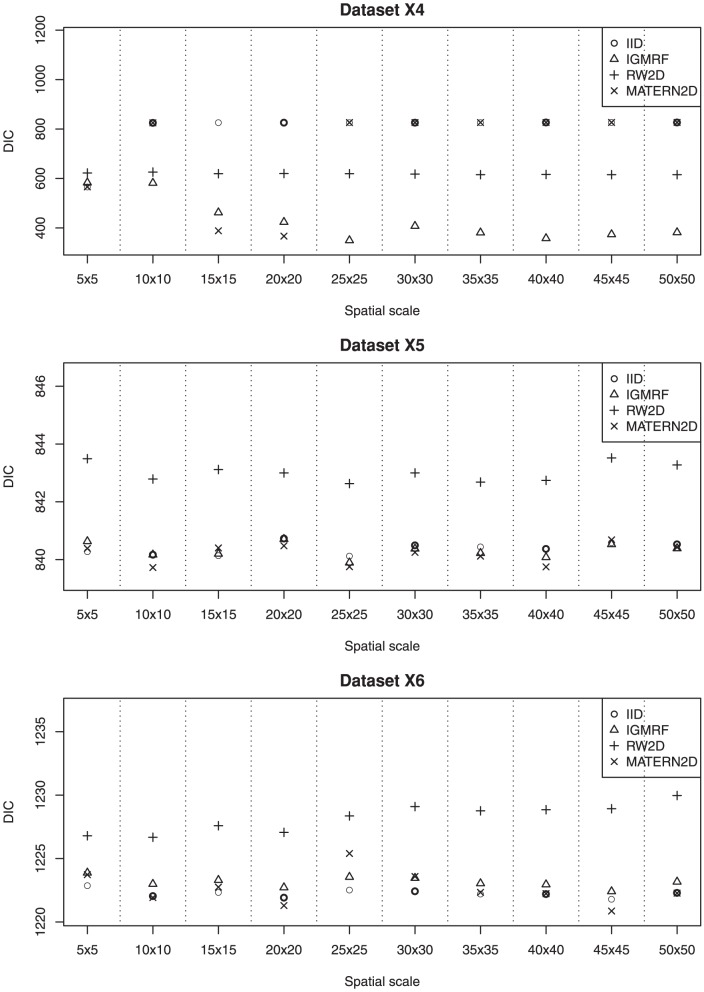
The estimated DIC of the four models for datasets X4, X5 and X6 at various spatial scales. The most appropriate spatial scale for fitting dataset **X4** is at the grid 20×20 using the MATERN2D model or the grid 25×25 using the IGMRF model. For datasets **X5** and **X6**, the IID, IGMRF and MATERN2D models perform well at most spatial scales.

**Figure 4:**
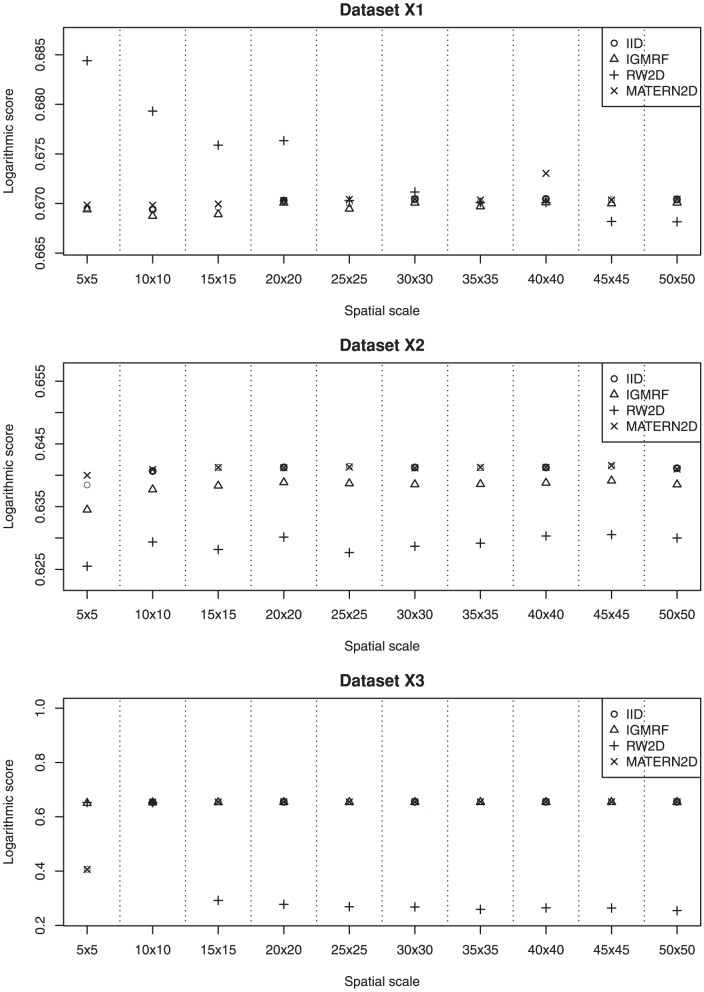
The estimated LS of the four models for datasets X1, X2 and X3 at various spatial scales. The RW2D model fitted at small grid cell sizes appears to be a reasonable choice for dataset **X1**. For dataset **X2**, the RW2D model produces the smallest LS at all spatial scales. The RW2D model also performs better than the three other models at grids 15×15 and above.

**Figure 5:**
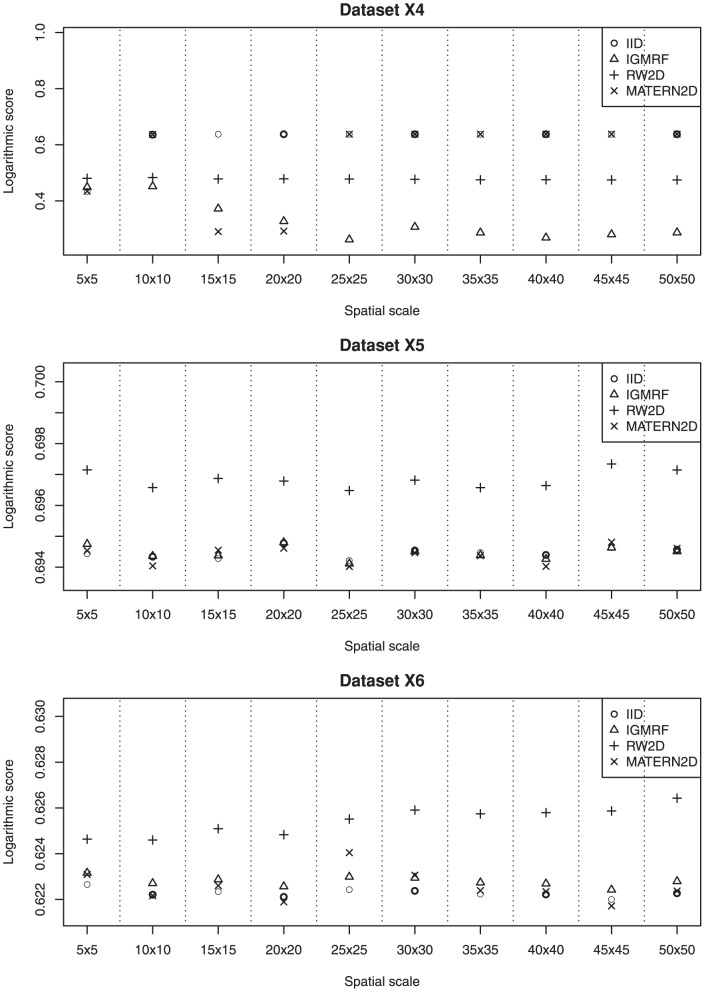
The estimated LS of the four models for datasets X4, X5 and X6 at various spatial scales. The most appropriate spatial scale for fitting dataset **X4** is at the grid 20×20 using the MATERN2D model or the grid 25×25 using the IGMRF model. For datasets **X5** and **X6**, the IID, IGMRF and MATERN2D models perform well at most spatial scales.

We describe the results for model fitting on the six simulated datasets and the realistic example in this section. The DIC for fitting the four models on the six simulated datasets at various spatial scales are presented in Figure 2 and Figure 3. Figure 4 and Figure 5 present the LS for fitting the four models on the six simulated datasets at various spatial scales.

### Dataset X1

The IID, IGMRF and MATERN2D models perform quite similarly at all spatial scales; whereas the RW2D model has larger DIC and LS than the other models at the first four spatial scales but its performance gradually improves as the grid cell size decreases. Across the spatial scales from the largest grid cell size to the smallest grid cell size, it is observed that the performance of the IID, IGMRF and MATERN2D models is fairly consistent. However, the RW2D prior appears to perform increasingly well as the grid cell size reduces. Therefore, for point data that are sparse and inhomogeneously distributed across the space such as dataset **X1**, the RW2D model seems to be a reasonable choice when fitted at small grid cell sizes. The results also suggest the need to repeat the analyses at multiple scales given the sensitivity of the models to changing spatial scales.

### Dataset X2

Based on the results obtained for dataset **X2**, the RW2D model produces the smallest DIC and LS at all spatial scales. It appears that the scores produced by this model are quite similar at all spatial scales, suggesting that the changes in grid cell size do not affect the model performance. The performance of the other three models is inferior to the RW2D model and is rather consistent across the spatial scales. We note that the point data in this dataset are sparse and distributed with the presence of local repulsion and mild inhomogeneity. The results suggest that the changes in the spatial scales do not affect the model outcomes for this spatial pattern.

### Dataset X3

In dataset **X3** which contains clustered cases and inhomogeneously distributed controls, the results clearly show the improvement in model fit and predictive performance for the RW2D model as the grid cell size decreases based on the decreasing DIC and LS. The RW2D model yields better fit than the three other models at grids 15×15 and above. A great improvement is seen for the RW2D model from the grid 10×10 to the grid 15×15. We note that at the grid 10×10, the number of individuals in the grid cells is highly varying with a maximum of 26 and median of 3.5, while less extreme at the grid 15×15 with a maximum of 17 and median of 2. There is a lot of inhomogeneity across the grid cells at the grid 10×10 which cannot be effectively smooth out by all four models. However, the disretization at grids 15×15 and above produces less inhomogeneity across the grid cells and thus the RW2D prior is able to smooth out the clusters more effectively. This is due in part to the ability of the RW2D prior in taking into account the first and second-order neighbors in spatial smoothing. For the clustered dataset presented here, there seems to be a need to discretize the study region into fine grid cells and the RW2D prior appears to be the most appropriate choice for spatial smoothing.

### Dataset X4

Dataset **X4** contains relatively small clusters of cases as compared with dataset **X3**. The results suggest that the most appropriate spatial scale for fitting this dataset is at the grid 20×20 using the MATERN2D model or the grid 25×25 using the IGMRF model. Too large or too fine grid cell sizes impair the model performance as shown by the MATERN2D model. The MATERN2D and IGMRF priors both appear to be good choices for the spatial smoothness priors for this dataset. However, the spatial scales should be chosen with caution as they affect model outcomes substantially.

As suggested by the results, some models behave differently at different scales, e.g., working relatively well at certain scales but not others. This could be related to the fact that the smoothness priors perform in different mechanisms at various scales due to the impact from the neighboring grid cells. For illustration, we present the estimated precision parameters for the MATERN2D model at various spatial scales in Table 1. It is shown that the mean and standard deviation of the precision parameters for 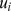 and 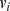 at grids 5×5 and 10×10 are very different from grids 15×15 and above, hence resulting in the varying DIC seen for the MATERN2D model in dataset **X4** (Figure 3). The performance of the MATERN2D model is very similar at grids 15×15 and 20×20 due to their similarity in the precision parameters. We note that at different scales, various degree of inhomogeneity across the grid cells is observed. When there is large inhomogeneity across the cells, a higher degree of spatial smoothing is imposed while less smoothing when there is small inhomogeneity. The change in the degree of spatial smoothing results in the changes in the precision parameters.

**Table 1:** The estimated precision parameters of the MATERN2D model at various spatial scales for dataset X4.

Spatial scale	Precision for 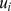 ( 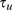 )		Precision for 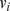 ( 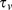 )	
	Mean	Std dev	Mean	Std dev
5×5	0.455	0.092	172.474	44.838
10×10	0.217	0.043	23.700	12.438
15×15	46.034	4.236	0.084	0.007
20×20	49.972	5.169	0.065	0.006
25×25	40.582	3.022	0.041	0.003
30×30	41.473	3.413	0.051	0.004

### Datasets X5 and X6

Datasets **X5** and **X6** contain point data that are inhomogeneously distributed across the space (similar to dataset **X1**). They are both denser than dataset **X1** and dataset **X6** is denser than dataset **X5**. The results for both datasets show that the IID, IGMRF and MATERN2D models produce good fit to both datasets at most spatial scales. The RW2D model is shown to yield the worst fit to the data across the spatial scales in both datasets. For both datasets, it appears that all the models perform rather consistently at all spatial scales. As a result, we note that the spatial scale is not an issue for dense point data and either one of the IID, IGMRF and MATERN2D models could be used in modelling.

### Humberside data

In order to understand the discretization of the Humberside dataset better, we provide the summaries of the number of events included in the grid cells for all the non-zero cell counts at the different spatial scales (Table 2). The maximum number of cases decreases from 33 at the largest grid cell size to four at the smallest grid cell size while the maximum number of controls decreases from 83 at the largest grid cell size to nine at the smallest grid cell size. It is shown that the number of events contained in the grid cells has only slight differences for the grid 30×30 up to the grid 50×50, which suggests that the grid 30×30 might be a suitable scale and a finer grid cell is not required.

**Table 2:** Summary of the number of events in the grid cells for all the non-zero cell counts at various spatial scales for the Humberside dataset.

Spatial scale	Case			Control		
	Min	Mean	Max	Min	Mean	Max
5×5	1	6.20	33	1	11.75	83
10×10	1	4.77	22	1	6.13	44
15×15	1	2.82	10	1	4.15	29
20×20	1	2.95	11	1	3.62	17
25×25	1	2.39	8	1	3.36	14
30×30	1	1.88	4	1	2.77	10
35×35	1	2.00	6	1	2.71	12
40×40	1	1.77	5	1	2.31	11
45×45	1	1.68	4	1	2.14	8
50×50	1	1.55	4	1	2.10	9

**Figure 6:**
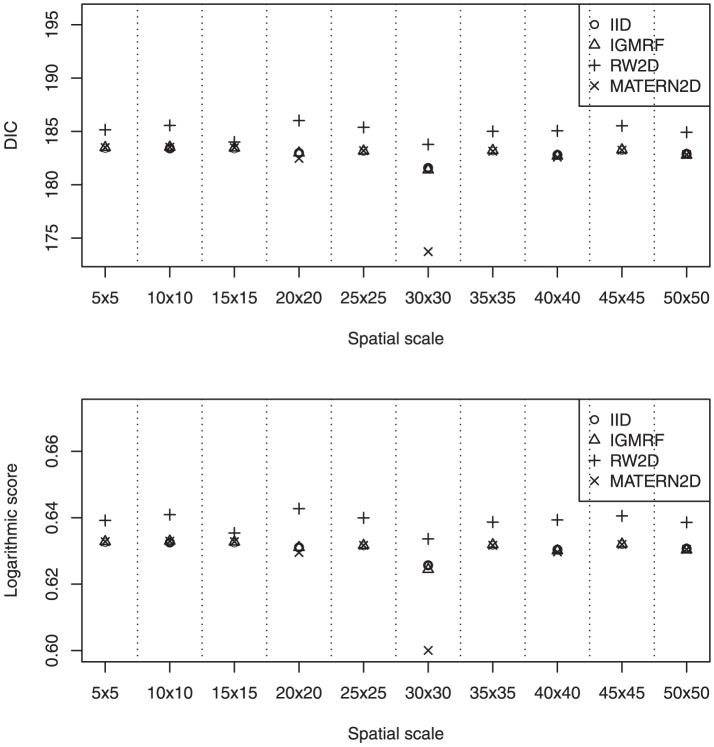
The estimated DIC and LS of the four models for the Humberside dataset at various spatial scales. The dataset should be fitted at the grid 30×30 using the MATERN2D prior for spatial smoothing.

Based on the results obtained from modelling the Humberside dataset, the IID, IGMRF and MATERN2D models produce similar DIC and LS at the various spatial scales (Figure 6) but the RW2D model performs slightly worse than the other three models at all instances. At the grid 30×30, a slight improvement is observed for all the models. Also, at this scale, the MATERN2D model outperforms the other models substantially. It could be seen that the performance of the MATERN2D model is rather sensitive to the changes in the spatial scales. In short, the results suggest that the dataset should be fitted at the grid 30×30 using the MATERN2D prior for spatial smoothing. Table 3 summarizes the results for fitting the models at various spatial scales for various datasets described above. The implications of these results are considered further in the Discussion.

**Table 3:** Summary of results for fitting the four models at various spatial scales for various spatial patterns.

Spatial patterns	Recommended spatial smoothness priors and spatial scales	Sensitivity of the models towards the changing spatial scales
Sparse inhomogeneous point pattern (dataset **X1**)	The RW2D model at small grid cell sizes.	The IID, IGMRF and MATERN2D models perform consistently at all spatial scales; the RW2D model is sensitive towards the changing grid cell sizes.
Sparse point pattern with local repulsion and mild inhomogeneity (dataset **X2**)	The RW2D model; spatial scales have little impact on model outcomes.	All four models perform rather consistently at all spatial scales.
Sparse inhomogeneous point pattern with large clusters (dataset **X3**)	The RW2D model at grids 15×15 and above.	The IGMRF model performs consistently at all spatial scales; the IID, RW2D and MATERN2D models are sensitive towards the changing grid cell sizes.
Sparse inhomogeneous point pattern with small clusters (dataset **X4**)	The MATERN2D model at the grid 20×20 or the IGMRF model at the grid 25×25.	The RW2D model performs consistently at all spatial scales; the IID, IGMRF and MATERN2D models are sensitive towards the changing grid cell sizes.
Dense inhomogeneous point pattern (datasets **X5** and **X6**)	The IID, IGMRF and MATERN2D models; spatial scales have little impact on model outcomes.	All four models perform rather consistently at all spatial scales.
Sparse point pattern with clusters (the Humberside dataset)	The MATERN2D model at the grid 30×30.	The IID, IGMRF and RW2D models perform rather consistently at all spatial scales; the MATERN2D model is sensitive towards the changing grid cell sizes.

## Discussion

We evaluated the performance of a range of spatial smoothness priors (an intrinsic Gaussian Markov random field (IGMRF), a second-order random walk on a lattice (RW2D), and a Gaussian field with Matérn correlation function (MATERN2D)) and spatial scales for various spatial structures using deviance information criterion (DIC) and logarithmic score (LS). The simulated datasets consist of points that are distributed across the space at various spatial patterns. The Humberside data are real phenomena where the data points are spatially sparse and exhibit a cluster. The results in this study suggest that different spatial smoothness priors and spatial scales may be appropriate for different patterns of spatial point-based data.

We note that for spatially sparse and inhomogeneously distributed point pattern (dataset **X1**), our study shows that it is necessary to include a spatially structured component, in addition to the unstructured component, to the model. The RW2D model at small grid cell sizes is an appropriate choice of modelling as it is the most parsimonious model (based on the DIC) and has the best predictive performance (based on the LS). Dataset **X5** and dataset **X6**, which are essentially similar structures to dataset **X1** but in a denser pattern, do not produce similar results to dataset **X1**. The results suggest that when denser point data are involved, the changes in spatial scales have little impact on the model outcomes. In addition, the spatial effect does not necessarily have to be included in the model as the unstructured component alone suffices. However, if desirable, the IGMRF and MATERN2D priors may be used as priors for the spatial effect as they do not impair the model performance.

When the point patterns with local repulsion and mild inhomogeneity (dataset **X2**) are modelled, the spatial component should be included and assigned the RW2D prior, in addition to the inclusion of the IID component in the model, as suggested by the results. Nevertheless, the spatial scales do not seem to matter in modelling this sort of spatial structure where the points are distributed across the space with local repulsion and mild inhomogeneity. For the inhomogeneous point pattern with a few large clusters (as portrayed by dataset **X3**), the RW2D model at fine grid cell sizes is shown to be a good modelling choice.

The sparse inhomogeneous point pattern with a number of small clusters across the space (dataset **X4**) appears to be quite sensitive to the changes in the spatial scales. As shown in the results, the MATERN2D model at the grid 20×20 and the IGMRF model at the grid 25×25 are the two appropriate modelling approaches for this point pattern. The model performance (based on the DIC and LS) becomes worse when larger or smaller grid cell sizes are used in modelling. This has addressed the need to select the spatial scales with caution when complicated spatial structures are of interest.

The realistic example studied here (the Humberside dataset) has further confirmed that for sparse point pattern with potential spatial clustering, the spatial scale and spatial smoothness prior have to be chosen carefully in modelling. The model fit (as guided by the DIC) and predictive performance of the models (as guided by the LS) differ at the different spatial scales. The results for this dataset show that the best modelling approach for this dataset is the MATERN2D model at the grid 30×30. This complements the results for dataset **X4**, in which both of these sparse datasets with clustering appear to be quite sensitive to the changes in the spatial scales. Furthermore, the MATERN2D model is shown to be a good modelling approach for both datasets.

The various spatial smoothness priors considered in this study have been shown to be applicable for different spatial structures. We note that it is possible to choose the appropriate prior based on the spatial structures but a range of priors should generally be considered. As suggested by our study, the RW2D prior is a reasonable choice for spatial smoothing when spatially sparse point patterns are involved, regardless of whether the points are homogenous or inhomogeneously distributed across the space. The RW2D prior imposes spatial smoothing by taking into account the first and second-order neighbors. Our study also shows that the IGMRF prior is suitable for spatial smoothing in spatially dense and inhomogeneous point patterns as it considers only first-order neighbors. The RW2D prior is essentially a second-order IGMRF on a lattice. It is quite flexible due to its invariance to addition of a linear trend. The RW2D prior imposes a higher level of spatial smoothing than the IGMRF prior due to the presence of the second-order neighbors. Sparse data need more spatial smoothing than dense data, therefore the RW2D prior works well in this context. If spatially dense and homogeneous point patterns are considered, the model may not include the spatially structured component but only the unstructured component assigned the IID prior. The MATERN2D prior appears to be well-suited for capturing the spatial effect in spatially clustered point patterns but it is very sensitive to the changes in spatial scales. This could be due to the representation of the smoothness parameter which gives the model great flexibility in modelling clustered point data that require a relatively high level of spatial smoothing.

In conclusion, we note that it is crucial to repeat the spatial analyses at multiple spatial scales when modelling inhomogeneously distributed point patterns as the model fit and predictive performance of the models appear to vary at different spatial scales. Methods for testing spatial heterogeneity such as Tango's Index [42] and Moran's I [43] could be used to decide if a given spatial dataset is inhomogeneously distributed. Inspection of detailed plots of the spatial data may also be a good guide to examine the presence of spatial inhomogeneity. For the inhomogeneous point patterns that do not contain clusters, the model performance improves as the grid cell size reduces. For the inhomogeneous point patterns that contain clusters, the appropriate spatial scale can be chosen by repeating the analyses at a range of spatial scales. On the other hand, the spatial scales appear to have little impact for homogeneously distributed point patterns. Also, it may not be necessary to include the spatially structured component in modelling of homogeneous point patterns unless desirable.

An acknowledged limitation of the study is that we simulated one scenario for each point process structure of interest. Therefore, we are reserved about the generality of the conclusions drawn above. For future work, more than one simulation scenario for a continuum of point process models with varying spatial structures could be studied in order to achieve more general conclusions. In this study, we consider grid cells with equal sizes as it was argued by [44] that in the specification of neighborhood structure, all regions should be of similar size and arranged in a regular pattern. For regions with different sizes, possible neighborhood structure definitions are some known function of the distance between centroid of areas [45]; an intrinsic conditional autoregressive (ICAR) prior [15] using weight definition, and alternative specifications within the CAR class [46]. Further investigation could be carried out to examine the impact of the changes in the shape of the regions including regular and irregular sizes.

Given the different results observed and different inferences made at the different spatial scales, it is crucial to repeat the analysis at different scales as the data may contain useful information at more than just one scale. It is also important to take into account the spatial scale that is of interest in a particular problem, i.e., the scale at which decisions or inferences will be made in practice. Often, disease management and policy making of subpopulation require modelling at a coarser scale than that required for understanding individual influences or associations. The choice of spatial scale is typically influenced by geo-political considerations, for instance, administrative districts are often used to describe and to understand geographical variation of a disease, with the aim being to assist public health decision making. Similarly, the identification of population-based clusters may differ from local clusters, with different interpretations and decision/action implications.
